# Comparison Between Family Function Dimensions and Quality of Life Among Amphetamine Addicts and Non- Addicts

**DOI:** 10.5812/ircmj.9947

**Published:** 2013-04-05

**Authors:** Mohammad Eshagh Afkari, Afsaneh Ghasemi, Davoud Shojaeizadeh, Azar Tol, Abass Rahimi Foroshani, Mohammad Hossein Taghdisi

**Affiliations:** 1 Department of Education and Promotion, School of Public Health, Tehran University of Medical Sciences, Tehran, IR Iran; 2 Department of Epidemiology and Biostatistics, School of Public Health, Tehran University of Medical Sciences, Tehran, IR Iran

**Keywords:** Employee Performance Appraisal, Quality of Life, Behavior, Addictive, Amphetamine

## Abstract

**Background:**

One of the most important factors in drug abuse and drug avoidance is family and its function.

**Objectives:**

This study aimed to compare family function and quality of life dimensions among Amphetamine addicts and non-addicts.

**Materials and Methods:**

The current study is a case-control, which assessed 95 Iranian addicts and 95 non-addicts. Sampling method in the addicts group was random clustering. The non-addicts were selected from accompanied addicts in other centers with respect to the demographic characteristics. The instruments were Family Assessment and Quality of Life (SF-36) scales. SPSS software version 11.5 was used for statistical analysis and Pearson’s correlation coefficient, stepwise regression analysis, and independent samples t-test were conducted.

**Results:**

The study revealed that some disorders in family function dimensions were higher in the addicts compared to non-addicts. Addicts have a quality of life lower than non-addicts (P < 0.05). There was a relationship between different dimensions of family function and the quality of life in both the addicts and non-addicts (P < 0.05). Regression analysis showed that roles dimensions and family function could roughly account for 17% of the changes in the addicts’ quality of life while in the non-addicts, behavioral control dimension of family function could account for roughly 17% of the changes in their quality of life.

**Conclusions:**

Regarding the study findings, there was a significant difference between family function dimensions and quality of life among addicts and non-addicts.

## 1. Background

Drug abuse is one of the most dangerous phenomena in human societies in the present era. Today, addiction is so widespread in the world that it has turned into a chronic and social disease and has threatened social security. According to formal data, 1.2 to 2 million of Iranians are addicts to Amphetamine with the average age of 18. However, 11 million of the country's population encounters an addiction problem for themselves or for their family members and friends (1). Within the past 30 years, Iran has faced an 8% growth in the annual rate of addiction (2) .This appears to be a critical issue since it influences the young generation and will result in depersonalization and weakness of physical ability and the youth’s detachment from the active sphere of life. The number of adolescents turning to drug abuse is increasing, and to control it, new techniques should be employed by prevention and treatment programs (3, 4). There are different reasons explaining people's tendency towards drug abuse: Some people are seeking to be accepted by the society and some others are trying to appear more grown up (5). Society and acquaintances also play a part in the increased abuse of drugs. In some social groups, drug abuse is a prerequisite for being accepted by others. This is supported by studies, which report that drug abuse suggestion by friends leads to the increased abuse of drugs (6). Jenaabadi et al. (2011) argues that those who participate in instructional courses encounter fewer problems concerning peer pressure being the cause of drug abuse of different kinds. The emotional consequences of drug abuse make the addict feel strong and secure towards pressures, impulses, and the issues ahead. Some studies suggest that teaching life skills to students can result significant changes in their knowledge, attitude and self-esteem (7). In a study on 400 patients who were under treatment for heroin and other opioids, it was found that the inability of anger management was the most salient emotional pattern in these people (8). In addition, according to the World Health Organization (WHO), quality of life is "people's perception of life conditions in terms of the culture and value system, which is concerned with goals, expectations, criteria and important affairs" (9).

Quality of life includes two dimensions, psychological and physical functioning and both are harmed with drug addiction because of the negative psychological (e.g. depression, anxiety, and disruption of family relations) and physical (e.g. bodily pain, and physical weakness) consequences of addiction. Studies have indicated that physical and psychological consequences of addiction result in a decrease in quality of life and life satisfaction of the addicts (10, 11). Addicts have different biological, psychological, social and emotional requirements, which are different from those of healthy people (12). One of the most influential factors of 60th drug addiction and drug withdrawal is family and family function. Family passes on values, improves morality and refines behavior. Studies have shown that there is a significant relationship between disorder of family function and anti-social behavior, aggression and addiction. There are different types of family conflicts. The two most important dimensions of family conflicts influential in increased drug abuse are a lack of relationship and inability of solving family problems (13). Family function is concerned with the way family members relate to each other, their interaction and maintenance of relationships, the way they decide and solve the problems (14). In other words, family function is an important aspect of the household environment and it influences the children's physical, social and emotional health. In fact, what occurs within a family and the way the family functions can be a key factor in developing flexibility and decreasing current and future dangers related to undesirable events and inappropriate conditions. Motivating and fostering environments enables children to learn and promote. In contrast, dysfunctional family environments can be very harmful for a large number of children's developmental dimensions and their positive transformation to adulthood (14). Family problems can result in educational underachievement, disorder in social relations, social seclusion, and alcohol and drug abuse (15). Since a family has a pivotal role in the socialization of the adolescents, family circumstances have been found to be among the main risk factors for an adolescent's drug abuse. Drug abuse is associated with a malfunction in parental roles, parent-child conflicts, intra-family conflicts, parent’s acceptance of the adolescent's drug abuse, and alcohol and cigarette use by parents, at home. A weak communicative functioning of the family is also reported to be associated with the adolescent's addiction. Parents' inadequate supervision and discipline is usually a strong predictor of drug abuse in adolescents (16). A study by Yen et al.’ (2007) showed that elements such as extensive conflicts between parents and children, alcohol use by the siblings for fun, the positive attitude of parents towards the adolescent's drug abuse and the inappropriate family function can be among the main predictors of internet addiction. Moreover, the first three mentioned factors could predict drug abuse (16). Searight et al. (1991) found that families with drug abuse had more restrictions for expressing feelings and thoughts, had less respect for private boundaries, less trust in others, had a more negative and critical environment in the family and felt emotional distance as compared to the non-clinical samples (17). Another research by Agha et al’ (2008) showed that there was a significant difference in the psychological problems and family function of addicts and non-addicts. Family can provide children with incentives, which influences their physical and mental development (13). The parent-child relationship of the non-addict adolescents in all its dimensions was of a higher quality than for the addict adolescents (18).

## 2. Objectives

Due to the necessity of this issue, this study was conducted to compare family function and quality of life dimensions among Amphetamine addicts and non-addicts in Iran.

## 3. Materials and Methods

The population of this case control research consisted of all of the addicts who had referred to The Drug Addiction Withdrawal Centers of Tehran University of Medical Sciences, Iran during 2012. This study was approved by the Deputy of Research and Ethics. Furthermore, it was approved by the Iranian Registry of Clinical Trials (10604) and ethical points were approved by Tehran University of medical Sciences (17090). Based on the available information and experience, uneducated addicts have a poor quality of life in Iran and we guess that they make up 50% of the total number of addicts in Iran. Thus, sample size was set so that if in later stages of this study, education was found to modify quality of life variable, or of moderate and desirable quality of life in educated addicts was 2.5 times versus not educated addicts with 95 present confidences and 80 percent power was significant, we used following formula. P = 0.5, P1= 0.7, 0.6, N = 95 in each group. The research sample included 95 addicted and non-addicted individuals. The sampling method used was randomized clustering in to two groups. At first, 4 centers out of 9 were randomly selected. Next, 95 individuals were randomly selected from these four centers. The sample's age ranged from 19 to 45 years and their educational degrees varied from elementary to bachelors. Also, this strategy was conducted in the control group but in another five centers. Eligible participants of the study were addicts and non-addicts referred to The Drug Addiction Withdrawal Centers of Tehran University of Medical Sciences; addicts who used amphetamine were placed in the case group and non-addicts who did not use drugs and had accompanied addicts were in the non-addicts group. Through randomization, participants of each group were selected if they met the inclusion criteria with respect to addiction and had provided the written informed consent. In order to control Berkson’s bias, we selected the cases from four centers while the control group was randomly selected from non- addicts’ of the other five centers.

Instruments used for this study were as follows:

1. Family Assessment Device (FAD): This questionnaire included 60 items constructed for the assessment of family function by Epstein et al. (1983). Each sentence was given a score in the range of one to four. The negative sentences were scored as follows: completely agree = 4, agree = 3, disagree = 2, and completely disagree = 1. Positive sentences were scored conversely. A high score indicates family malfunction (19) . Reliability was tested by the test-retest method. Total Chronbach's alpha was 94% for the whole questionnaire. Chronbach's alpha of the subscales of general functioning, problem solving, relation, roles, emotional accompaniment, behavior control and emotional association were 0.78, 0.72, 0.70, 0.71, 0.73, 0.66 and 0.71 respectively (20). In the present study, the Chronbach's alpha in total (FAD) was calculated to be 0.92.

2. SF-36: This Questionnaire is a good measure of individuals' perception of their wellbeing. It consists of 36 items measuring eight subscales related to wellbeing: physical functioning, physical roles, bodily pain, social functioning, emotional roles, general health, liveliness, and mental health. In general, the questionnaire evaluates both physical and mental approaches. It has been shown that this questionnaire has high validity and reliability. Validity and reliability of a questionnaire was evaluated by Montazeri et al. (21). From ethics point of view no one was forced or obliged to participate in the study and the patients’ privacies were respected. All participants filled written informed consents.

### 3.1. Statistical Analysis

The SPSS software version 11.5 was used for statistical analysis. Pearson’s correlation coefficient, stepwise regression analysis and independent samples t-test was conducted. Kolmogorov-Smirnov test was performed to test normalization of data, which was approved (P > 0.05). Results were considered significant at the conventional P < 0.05 level.

## 4. Results

Table 1 shows that in all dimensions of family function, the mean scores are different in the addicts and non-addicts and that for all dimensions, the mean scores are higher in the addicts than in the non-addicts. However, a higher score in the FAD indicates malfunction. Therefore, it can be concluded that addicts have a higher degree of malfunction in family performance. To investigate the mean difference between the two groups in terms of the quality of life, independent samples t-test was used. Table 2 shows that there is a significant difference between the two groups in terms of the mean scores for the quality of life and its dimensions and in all cases, the mean scores of the non-addicts are higher than those of the addicts. Therefore, the results suggest that the addicts have a lower quality of life. In order to investigate the relationship between family function and its subscales and the quality of life, Pearson's correlation test was used, for which the results are presented in table 3 for the two groups.

**Table 1.** Comparison of Family Function and its Dimensions Among the Study Population

Variables	Mean	Standard deviation	Mean difference	Standard error of difference	Degrees of freedom	t-test	P value
**Family function**			22.47	2.92	212	7.681	< 0.001
Addicts	144.96	17.49					
Non-addicts	122.49	24.68					
**Problem solving**			1.15	0.38	212	3.014	0.003
Addicts	12.63	2.79					
Non-addicts	11.49	2.77					
**Relation**			2.57	0.39	212	6.579	< 0.001
Addicts	14.08	2.80					
Non-addicts	11.51	2.90					
**Roles**			2.37	0.57	212	4.772	< 0.001
Addicts	22.56	3.13					
Non-addicts	20.19	4.07					
**Emotional accompaniment**			3.12	0.51	212	6.162	< 0.001
Addicts	19.73	3.52					
Non-addicts	16.61	3.87					
**Emotional association**			3.58	0.58	212	6.150	< 0.001
Addicts	20.60	4.03					
Non-addicts	17.02	4.46					
**Behavior control**			4.31	0.54	212	7.898	< 0.001
Addicts	22.27	3.66					
Non-addicts	17.96	4.29					
**General functioning**			5.39	0.79	212	6.777	< 0.001
Addicts	30.88	4.85					
Non-addicts	25.49	6.64					

**Table 2.** Comparison Between the Quality of Life and its Dimensions Among the Study Population

Variables	Mean	Standard deviation	Mean difference	Standard error of difference	Degrees of freedom	t-test	P value
**Quality of life**			-735.89	69.65	212	-10.565	< 0.001
Addicts	1969.58	519.24					
Non-addicts	2705.47	499.54					
**General ** **health**			-25.93	6.65	212	-3.90	< 0.001
Addicts	113.78	51.42					
Non-addicts	138.72	45.68					
**Activity limitation**			-211.68	29.25	212	-7.247	< 0.001
Addicts	680.37	250.45					
Non-addicts	892.06	168.79					
**Physical health**			-110.98	12.37	212	-8.971	< 0.001
Addicts	163.32	93.56					
Non-addicts	274.30	87.31					
**Mental health**			-75.23	10.16	212	-7.404	< 0.001
Addicts	117.76	74.61					
Non-addicts	192.99	74.03					
**Social activities**			-32.10	6.69	212	-47.799	< 0.001
Addicts	98.748	46.26					
Non-addicts	130.84	51.46					
**Pain**			-42.20	6.91	212	-6.102	< 0.001
Addicts	123.55	54.83					
Non-addicts	165.75	45.95					
**Energy and spiritual feelings**			-164.89	25.63	212	-6.431	< 0.001
Addicts	512.94	193.94					
Non-addicts	677.80	180.84					
**General health**			-82.94	9.45	212	-8.77	< 0.001
Addicts	213.78	72.70					
Non-addicts	296.73	65.24					

Table 3 indicates that there is a significant relationship between family function and all its dimensions and the quality of life of both the addicts and non-addicts, since a high score indicates family malfunction. Therefore, it can be suggested that there is a converse relationship between family function and the quality of life. In addition, as indicated by Table 3, a comparison of the correlations between the two groups shows that there is a higher correlation between emotional accompaniment, behavior control and general functioning, and the quality of life of the non-addicts than the addicts. In order to predict the quality of life of the addicts and non-addicts on the basis of family function dimensions, a stepwise regression analysis was used (Table 4).

**Table 3.** Correlation Between Family Function and its Dimensions and the Quality of Life in the Two Groups

Group	Variables	Family functioning	Problem solving	Roles	Relation	Problem solving	Emotional association	Behavior control	General functioning
**Addict**	Quality of life	-0.291	-0.284	-0.311	-0.362	0.222	-0.239	-0.212	-0.201
**Non-addict**	Quality of life	-0.382	-0.296	-0.322	-0.349	-0.250	-0.262	-0.431	-0.318

**Table 4.** Summary of the Regression Model of the Quality of Life Based on Family Function Dimensions for the Two Groups

Groups	Variables	R	Explanation coefficient	Adjusted R squared	Standard error of estimation
**Addict**	Roles	0.362	0.131	0.123	486.27
	Roles, Relation	0.414	0.172	0.156	477.09
**Non-addict**	Behavior control	0.413	0.171	0.163	

Table 5 indicates that in the first step, in the addict group, the Sub - variable of roles, which had a stronger correlation with the quality of life, entered the regression equation and accounted for 13.1% of the quality of life. At the second step, the variable of relation that came second for the strength of correlation with the quality of life entered the regression equation, and along with the variable of roles, they account for 17.2% of the variance of the quality of life. The variables of problem solving, emotional accompaniment, emotional association, general functioning and behavior control while being meaningful, could not be predictive and did not enter the equation since they had a weaker correlation with the quality of life. Moreover, in the non-addicts, at the first step, the variable of behavior control, which had a stronger correlation with the quality of life entered the regression equation and alone accounted for 17.1% of the changes in the quality of life. Other dimensions of family function could not be predictive and did not enter the regression equation.

**Table 5.** Regression Coefficients for Quality of Life Based on Family Function Dimensions for the Two Groups

Groups	Variables	B	Beta	Standard error	t	P value
**addict**	Roles	-59.931	-0.362	15.05	-3.983	< 0.001
	Roles	-51.549	-0.311	15.23	-3.378	< 0.001
	Relation	-38.491	-0.208	17.08	-2.253	0.02
**Non-addict**	Behavior control	-48.053	-0.413	10.34	-4.647	< 0.001

## 5. Discussion

The present research aimed to determine the relationship between family function and the quality of life and compared this between addicts and non-addicts. The research results showed that there is a relationship between family function dimensions and the quality of life of the addicts and non-addicts. There is a negative relationship between the malfunction of family relations and the children's quality of life. The results of the present research are in accordance with the findings of the Christen study (19) who investigated the relationship between family efficacy and children's general health. He found that a better functioning of the family is related to a higher mental health. In addition, the present study is compatible as it investigated the relationship between communicative patterns of family relations and the quality of life. The results showed that the family's conversational orientation is a positive and meaningful predictor of the quality of life, and its conformity orientation is a negative and meaningful predictor of the quality of life. Also, the current study indicated that the dimensions of family function and the quality of life are different in the addicts and non-addicts. In other words, the addicts have a lower mental health than the non-addicts. This finding is in accordance with the results of the Agha et al.’ study (13) who indicated that there is a significant difference in the psychological and functioning problems of families of addicts and non-addicts. They found that addicts have a lower mental health as compared to the non-addicts. Searight et al. (1991) observed that families with drug abuse had more restrictions for expressing feelings and thoughts, had less respect for private boundaries, less trust in others, had a more negative and critical environment in the family and felt emotional distance as compared to non-addicts’ families (17). An appropriate family function is necessary for the wellbeing of an individual, family and society. A family with appropriate function can fulfill the emotional, mental and physical needs of its members. However, a family with an inappropriate function fails to fulfill these needs of its members. Failure to fulfill the family members' needs in different fields can influence their physical, social and emotional health. Various studies have shown that in families in which relationships between the members and the inter-family interactions are based on closeness, intimacy and agreement, all members are relatively resistant and immune from the life pressures. Ineffective people resort to drug abuse to decrease their problems; lack of emotional competence, inappropriate emotional skills, temporary relations, and inadequate ability in resolving conflicts can result in drug abuse (20). The results of the present research showed that there is a significant difference between the addicts and non-addicts in terms of their quality of life; this finding is in accordance with the findings of other studies (11, 12). To delineate this finding, it is argued that drug abuse has undesirable physical, mental and social consequences such as muscular weakness, bodily pain, lack of appropriate social relations, aggression, depression, anxiety, and an inadequate quality of life and life satisfaction. By making a change in behavior, self-esteem, nutrition, job and social relations, addiction in general changes the natural life of an individual, which in turn decreases his/her quality of life. Addicts lack the initiative to control the environment as related to others. Furthermore, their physical energy, life expectancy and life satisfaction will decrease (21). Numerous researches in Iran and other countries indicate the low quality of life of addicts because, in most cases, addiction has a negative influence on the quality of life (22). Therefore, since the quality of life of addicts’ families has many deficiencies, the basic problems of the family are intensified, and as well as having destructive effects on the addicts themselves, others such as their acquaintances and relatives are also vulnerable. Moreover, the addicts have a lower mental health than the non-addicts (23). Addiction is not an issue that develops momentarily and in the absence of the required conditions and factors. Recent clinical findings indicate that unhealthy conditions for the development, inclination and promptness of addiction are critical factors in the formation of addiction. Family as an important developmental context, which can create the inclination and promptness for addiction in its members. In the families in which there is contempt, threat and blame, there is no appropriate relation based on trust, the boundaries between family members are not clear and transparent, the roles and duties of the members are not compatible with their developmental stage, problem solving is not adequate, there is no emotional accompaniment, and there is no appropriate resolution for conflicts; all of these can be appropriate provisions for addiction. According to the results of the present research, it is suggested that basic preventive measures for addiction start with families. Providing families with instructions on the methods of effective communication, problem solving approaches, conflict resolution, accountability, understanding of feelings and emotions, and appropriate styles of child rearing can be effective. Families should participate and cooperate in the medical measures for the treatment of addiction and a change should be made in the entire system of the family. One of the reasons why all of the family members should receive support and services is the systemic nature of families. In a system, one section can influence all other sections and in turn be influenced by them. If addiction treatment is done without making any change in the system of the family, there would be a high possibility of addiction return since it is supposed that the family system plays an important part in the addiction of the individual, and if he/she returns to the same family with the same function, the change will not last for long. Therefore, both the prevention and treatment of addiction should start with the family.
